# Books Reviewed

**Published:** 1866-09

**Authors:** 


### Books Reviewed.

A Practical Treatise on Urinary and Renal Diseases, including urinary deposits, illustrated by numerous cases and engravings. By WILLIAM ROBERTS, M. D., Fellow of the Royal College of Physicians, London; Physician to the Manchester Royal Infirmary; Lecturer on Medicine in the Manchester School of Medicine. Philadelphia: Henry C. Lea, 1866.

Pathologists, chemists and physiologists, have recently devoted much attention to diseases of the kidneys, composition of urine, microscopical appearances of urinary deposits, functions of the kidneys, and morbid changes observed to take place in their structure, in disease, as well as their minute anatomy and natural function in health. Any correct knowledge of the diseases of these organs is of comparatively recent date, except so far as some functional disturbances may have been observed and their influence observed upon the general system known. The organic diseases of these organs have been little understood until the observations of Bright, Prout, and more recently a host of others, who have directed attention to this class of diseases and shown the nature, causes, morbid changes, and effects upon the system generally of defective or suspended function in these glands.—The author has divided his work into three parts, the first showing the physical and chemical properties of the urine, the various alterations which it undergoes under different circumstances of health and disease, the methods of examining the urine for clinical purposes and the significance of the changes observed; the natural and microscopical appearances of urinary deposits are described and figured, together with the extraneous matters which accidentally find their way into the urine. The second part treats of urinary diseases—diabetes insipidus, diabetes melletus, gravel and calculus, and chylous urine. The organic diseases of the kidney form the third division of the work, and this is the most important and valuable. He says, "the present moment is not favorable for a lucid description of Bright's disease and its allies. Not only is there a wide difference of opinion as to the clinical grouping of the cases, but the researches of the last three years indicate a necessity for a complete revision of our previous notions regarding the minute anatomy and the functions of the kidneys. The observations of Henle, and of the numerous inquirers who have followed him, have shown that the course and structure of the uriniferous tubes are far less simple than has been hitherto supposed; and the experiments of Oppler, Perls, and especially of Zalesky, challenge the very basis of the current opinion of the functions of the kidneys, and send us back to the older view, that the special ingredients of the urine—urea and uric acid—are actually formed by the kidneys, and not merely seperated by them from the blood."

The author has embodied the views and opinions of the best authors upon urinary diseases with his own experience and practical knowledge of their nature, causes and treatment, and the work as thus comprised is a very valuable addition to the standard English literature upon these subjects.

The Beacon; or a Warning to Young and Old. By William M. Cornell, M. D. L.L. D.

"This book is not written for the medical profession," and consequently it will not be reviewed with that scrutiny it might otherwise receive. It represents self-abuse as oftentimes the cause of epilepsy, consumption, idiocy and insanity, and contains chapters upon hereditary transmission of disease, management of children, causes of nervous diseases, etc. It appears to be a sort of medico-moral treatise, illustrated by cases and enforced by "practical remarks," showing the evils of self-abuse, nowhere omitting the central truth that the author has cured a great many similar cases, and makes it a "speciality" to treat nervous diseases, which are shown to include all or almost all the maladies which prevail among mankind. He says: "All kinds of convulsions, everything connected with neuralgia, St. Vitus' dance, spasms, contractions and expansions of the muscles, consumption, etc., etc., all are the effects of nervous influence; and hence, most of our diseases may properly be said to be nervous." Our readers will see at a glance what limitless number of diseases receive the "special" attention of a physician who has devoted years to the study and treatment of only one class.

---

The Medical Register of the City of New York, for the year commencing June 1, 1866, published under the supervision of the New York Medico-Historical Society. GUIDO FURMAN, M. D., Editor. Edw'd O. Jenkins, 20 North William street, 1866.

The fourth annual edition of this valuable little work is promptly on our table. Although an immense amount of labor is necessary in compiling and arranging a book of this kind, the author has succeeded in furnishing a complete medical directory of the various Societies, Colleges and Hospitals, together with the residence and location of all the regular physicians both in New York and Brooklyn. To this is added the State Medical Society and most of the County Societies throughout the State.

The author says that "it had been his intention to furnish the name and residence of the officers and members of each and every Medical Society in the State of New York, but owing to neglect of answering circulars addressed to officers and members of every society he was unable to give all."

This book emphatically speaks for itself, for it is rare to find so much information contained in so small a space. It is a work indispensable to the physicians of New York and Brooklyn, and highly useful to the profession throughout the State.

---

On Spermatorrhœa. Its causes, symptomatology, pathology, prognosis, diagnosis and treatment. By ROBERT BARTHOLOW, A. M., M. D., Professor of Physics and Medical Chemistry in the Medical College of Ohio; Lecturer on Clinical Medicine and Physician to St. John's Hospital, Cincinnati; formerly Assistant Surgeon (Captain) U. S. Army, etc., etc. New York: William Wood & Co., 61 Walker street, 1866.

Owing undoubtedly to the aversion which competent physicians entertain towards so disagreeable and to a certain degree disreputable subject as spermatorrhœa, but few special treatises on its pathology and treatment of a reputable character have been placed before the profession, and it affords us great pleasure to call attention to such a judiciously conceived and well executed monograph as that of Dr. Bartholow.

The writer justly considers the disease essentially as a deranged functional state of the spinal system, and that the lesions of the prostatic portion of the urethra and seminal ducts as an accidental complication, in opposition to the long established views of Lallemand, who regarded as spermatorrhœa an inflammation of the prostatic portion of the urethra and the seminal ducts, for the treatment of which he introduced the porte-caustic. Dr. Bartholow rejects the practice of cauterization entirely, and would justify its use only in—

1st.—Those cases in which chronic inflammatory changes exist as a complication of spermatorrhœa, and

2d.—Those in which the moral effect of the application is desirable. In the medical treatment the author's efforts are directed to the improvement of the general health by the administration of tonics in combination with the anaphrodisiacs, among which he considers bromide of potassium as the best.

